# It’s crunch time: Exploring the sensibility of food textural acoustics for individuals with dysphagia

**DOI:** 10.4102/sajcd.v68i1.806

**Published:** 2021-06-30

**Authors:** Tasneem F. Karani, Mershen Pillay

**Affiliations:** 1 Discipline of Speech-Language Pathology, School of Health Sciences, University of KwaZulu-Natal, Durban, South Africa; 2 Speech and Language Therapy,Massey University, Auckland, New Zealand; 3 Department of Health Professions, Manchester Metropolitan University, Manchester, United Kingdom

**Keywords:** food acoustics, texture, perception, physiology, multisensory eating, dysphagia

## Abstract

**Background:**

Various fields of study have alluded to food textural, and its associated acoustic, properties (i.e. food textural acoustics). However, because of the challenging nature of the inclusion of acoustic properties in diet textural modifications in dysphagia (swallowing disorders), this construct has not been sufficiently considered in the field.

**Objective:**

To investigate the sensibility of food textural acoustics as a construct to understand eating for individuals with dysphagia.

**Method:**

The study design was based on qualitative evidence synthesis methodologies. This involved revised scoping review methods (peer-reviewed published articles from 1980 to 2020 over seven databases), with an adapted consultation phase through online focus group discussions with six world experts. The data was analysed using frequency and thematic analysis, and ideology critique.

**Results:**

A total of 11 articles were included in the revised scoping review analysis (seven research studies and four review articles). The analysis of these articles revealed a lack of diversity in geography, discipline and perspective exploring the construct of food textural acoustics. A total of three themes with three associated core arguments emerged from the revised scoping review and the consultation phase. These arguments highlighted (1) the need to study food textural acoustics because of its salience and pleasure responses, (2) possible methodological dilemmas in studying food textural acoustics due to the complexity of eating, and (3) considerations with regard to the approach and positioning adopted when studying the construct.

**Conclusion:**

Food textural acoustics may be a sensible construct to understand eating for individuals with dysphagia. As eating is a complex process, there is a need to challenge the methods we use when studying this construct of food textural acoustics. We hope that this article inspires researchers and practitioners to think differently by using textural, and its associated acoustic, properties as a way to reimagine dysphagia practice, especially for those from low- to middle-income contexts such as South Africa and Brazil.

## Background

Eating is one of the most multisensory human experiences (Spence, 2017, 2020). The visual system is involved in the selection of foods, and the olfactory and gustatory systems make sense of the smell and taste of the food. The proprioceptive system assists in identifying textures and shapes of foods, while the auditory system plays a role in the sounds of biting and chewing of foods (Spence, 2015; Verhagen, 2007). Practitioners in the field of dysphagia (swallowing disorders) primarily consider sensory stimuli such as chemical, tactile and thermal properties in the management of individuals with dysphagia (Steele & Miller, 2010; Steele et al., 2015). Despite the multisensory nature of eating, acoustic stimuli are not predominately used. This is due to the challenging nature of selecting the appropriate food textures, that have robust and consistently replicable acoustic properties, yet still safe for consumption for individuals with dysphagia. Auditory properties are also not sufficiently foregrounded in comparison to the remaining senses in other fields such as cognitive neuropsychology and are often regarded as the neglected or ‘forgotten flavour sense’ (Spence, 2015).

The coronavirus disease 2019 (COVID-19) pandemic has highlighted the vital role of the senses in eating. Anosmia and ageusia (i.e. loss of the sense of smell and taste) are symptoms of the COVID-19 virus, which can lead to diminished pleasure in eating (Coppin, 2020; Menni et al., 2020; Parma et al., 2020). Can one still eat and survive with the disabling effects of the loss of smell and taste? Regardless of the temporary loss of these senses, an individual may rely on the role of texture and its associated acoustic properties for their hedonic or pleasure response when eating. This is as a result of the notion of auditory salience. Evolutionarily, auditory salience has been understood as acoustic properties of food signifying freshness, pleasantness and enjoyment (Spence, 2015; Tunick et al., 2013; Vickers, 1983).

Indeed, textural properties and acoustic properties are intimately connected. For instance, the force applied to a texturally hard food sample when biting into the food results in the production of vibrations that are transmitted to the ear as a wave via two pathways, namely, air-conduction and bone-conduction (Duizer, 2001; Vickers & Bourne, 1976). We perceive these vibrations as sounds. For the purpose of this study, we will refer to the textural, and its associated acoustic, properties involved during eating as food textural acoustics. There are several fields of enquiry that have alluded to food textural acoustics. This includes the well-established fields of engineering and physical sciences, gastronomical sciences, cognitive neurosciences and dysphagia sciences (see Appendix 1 for a review of the established fields that have made reference to food textural acoustics). This undeniable marriage of texture and acoustics was first recognised within the fields of engineering and physical sciences with the establishment of the notion of acoustic rheology (Peleg, 2017; Zadeike, Jukonyte, Juodeikiene, Bartkiene, & Valatkeviciene, 2018). We have borrowed from these fields and have chosen to use the term food textural acoustics. Although it is well-known in the aforementioned fields that texturally hard foods produce auditory stimuli, for example, crispy or crunchy sounds, dysphagia studies of diet textural modifications tend to marginalise how texture and acoustic properties are closely connected. It is necessary to note that textural properties cannot be divorced from its acoustic properties when recommending the commonly used compensatory strategy of diet textural modifications in dysphagia rehabilitation (Cichero, 2018; Cichero et al., 2017; Swan, Speyer, Heijnen, Wagg, & Cordier, 2015).

Other fields that are more recent and burgeoning such as the multisensory human–food interaction (MSHFI) and multisensory eating frameworks have also alluded to food textural acoustics. The field of cognitive neuroscience has studied acoustic stimuli by foregrounding music and the environment and its influence on behaviour and perception (Callan, Callan, & Ando, 2018; Carvalho, Wang, Van Ee, & Spence, 2016; Höchenberger & Ohla, 2019; Kantono et al., 2018; Spence, 2015, 2017). However, there is still a gap present in the fields at large exploring the influence of acoustic stimuli on physiological responses. We have not sufficiently considered the physiological responses such as the notion of autophony. Autophony refers to how the body generates and responds to internal sounds, for instance, sounds produced when eating and hearing one’s voice and breathing (Harris 2015; Mabaso, Malinga, & Paruk, 2018; Tidball & Fagelson, 2018). The various fields that have alluded to this construct of food textural acoustics, discussed above, speak to its overall construct validity or sensibility. We have chosen to use the notion of sensibility because of the complexity and marginality of the construct that we are studying. We have defined sensibility with reference to sensible knowledge and the judgement of the plausibility of a concept (Strati, 2007).

This study is part of a larger cluster of research projects under the THRIVE programme. THRIVE is an acronym for ‘Tackling Hunger by Research and Innovation in Vulnerable Environments’ (Pillay, 2013; Pillay & Kathard, 2018). The THRIVE programme strives to reposition swallowing and feeding in a way that promotes transformative practitioners who are concerned with the food security and sovereignty of their patients. Individuals with disabilities such as those with dysphagia may encounter difficulty in accessing food that is affordable and safe for consumption (Pillay, 2013; Pillay & Kathard, 2018). This is prevalent particularly in low- to middle-income contexts such as South Africa and Brazil. Low-to-middle income contexts may present with limited access to healthcare professionals, resources and equipment, where one can only target and invest in the food provided to these individuals. There is a specific THRIVE project that is motivated to develop more novel dysphagia interventions (Pillay & Kathard, 2018). In this study as part of this project, we would like to propose a transformation of the diets for patients with dysphagia (refer to Appendix 2 for a history of the study that includes three phases). We would like to conceptualise alternative methods of developing foods that consider the multisensory nature of food and the potential therapeutic benefits of acoustic properties. This may drive us to connect diet textural modifications to food security from the perspective of food safety and the appropriate food textures. This includes developing and investing in more ‘sensory responsive’ foods that in addition to having a hedonic response, we hope that these foods will have a therapeutic benefit for individuals with dysphagia. It is vital to highlight that despite the documented research on the hedonic response to food textural acoustics, certain individuals may dislike or have unpleasant experiences to textural acoustic properties. Sensory aversions and misophonia (i.e. a negative emotional reaction and dislike triggered by particular sounds such as eating and breathing) may also occur in both typical and atypical individuals (Little, Dean, Tomchek, & Dunn, 2017; Palumbo, Alsalman, Ridder, Song, & Vanneste, 2018).

### Objective

To investigate the sensibility of food textural acoustics as a construct to understand eating for individuals with dysphagia.

## Methods

### Study design

The underlying theory of this study originates from qualitative evidence synthesis (QES) methodologies with regard to creating evidence for a form of knowledge and practice. Traditionally in healthcare, researching a theoretical basis of a construct and using the evidence from the scientific research may lead to the development of clinical practices (Dodd, 2007; Sackett & Rosenberg, 1995). Essentially, we are arguing that thoughts (theory) determine our practice (clinical practice). We used this broad QES methodologies framework in a way that engaged us reflexively as researchers. Reflexivity refers to providing ‘attention to the complex relationship between the processes of knowledge production and the various contexts of such processes, as well as the involvement of the knowledge producer’ (Alvesson & Sköldberg, 2009:8). This QES methodology study design involved scoping review methods by Arksey and O’Malley (2005) and Levac, Colquhoun and O’Brien (2010) with an optional adapted consultation phase through online focus group discussions (Colquhoun et al., 2017). According to Arksey and O’Malley (2005), the consultation phase can be used in a variety of forms depending on the study aim. In this case, we have adapted and expanded the consultation phase by further exploiting it and more closely engaging with expert practitioners in the field. This has been used to dialogue the experts’ opinions with the published literature and scoping review findings regarding the construct of food textural acoustics in eating for typical adults. Similar to other fields of enquiry, this information may be applied to individuals with dysphagia.

### Data sources

This study involved two main interconnected data sources: (1) published, peer-reviewed articles obtained via seven databases, detailed below, and (2) online focus group discussions with six world experts. We reviewed the published articles over a 40-year period (1980–2020). We also conducted focus group discussions with six world experts from several fields to gather their opinions on the construct of food textural acoustics in eating. This method will be expanded on in the next section. We hoped that this would inform a novel way of understanding the construct and its potential clinical application to the field of dysphagia. We have intentionally revealed the identities of the world experts, with their permission, to initiate a conversation and to develop a community of practice from a variety of fields regarding the construct of food textural acoustics.

### Data collection

We used the following methods to design the revised scoping review methodology; Preferred Reporting Items for Systematic Reviews and Meta-analyses extension for Scoping Reviews (PRISMA-ScR) framework (Schultz et al., 2018; Tricco et al., 2018), and guidelines by Joanna Briggs Institute (Moola et al., 2015) for the search strategy. These methods were used to systematically and comprehensively map concepts and the distribution across the fields (Levac et al., 2010). The revised scoping review included the following: (1) a pilot study that was conducted by an external reviewer to assist in planning the main study and analysing its validity (In, 2017), (2) a main study which involved a blind review by two reviewers to screen the titles and abstracts to ensure inter-rater consistency, and (3) conflict resolution through discussions between the two reviewers to finalise the selection of the full-text articles. Figure 1 depicts the parameters used in the revised scoping review.

**FIGURE 1:**
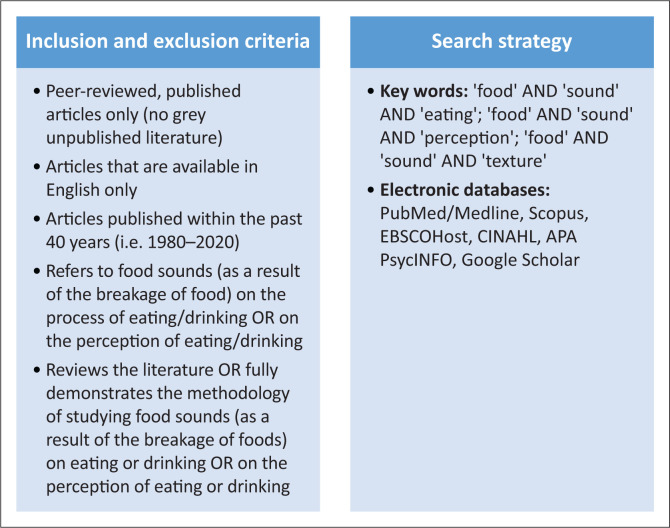
Parameters of the revised scoping review.

We consulted six world experts through online focus group discussions following the scoping review. The world experts represented various disciplines of study: (1) mechanical engineering (Associate Prof. Ben Hanson; University College London); (2) food science (Prof. Lisa Duizer; University of Guelph, Canada); (3) audiology (Prof. Wayne Wilson; University of Queensland, Brisbane); (4) cognitive neuropsychology (Prof. Massimiliano Zampini; University of Trento, Italy); (5) a sommelier, (Chef Ferran Centelles; Director of BulliPedia Drinks elBullifoundation, Barcelona), and (6) speech pathology and audiology (Associate Prof. Mershen Pillay; University of KwaZulu-Natal, South Africa). These experts were purposefully selected based on their respective fields of expertise and the knowledge of them contributing collaboratively and constructively to the development of the study. Pillay co-author of the article, was included in the study as he is an expert who has developed the THRIVE framework and provided a perspective from a low- to middle-income context on the matter. The expert consultations included self-developed semi-structured open-ended questions to promote dialogue between the experts. The questions focused on the experts’ opinions regarding food textural acoustics, its relevance to their respective fields and discussion relating to some of the published literature and scoping review findings. We also shared details of the methodology and results of phase 2 of our study. Our analysis yielded core constructs and considerations when studying food textural acoustics, which will be presented under the ‘Results and discussion’ section.

### Data analysis

We conducted two main data analyses: (1) frequency analysis and basic content analysis of the 11 included articles, and (2) thematic analysis of the 11 articles and the focus group discussions. We used *Mendeley* with *Covidence* for data management of the articles retrieved from the review. We identified the bibliographic coverage of the 11 included articles by conducting frequency analysis of author names, year, knowledge fields and affiliation (journal). We also conducted a basic content analysis to identify and tabulate the frequently occurring constructs, methodologies used and overall findings. The primary researcher transcribed the data verbatim from the consultation phase. We conducted member checking to improve the trustworthiness and validity of the data (Birt, Scott, Cavers, Campbell, & Walter, 2016). This was performed by sending out the audio recordings and transcripts of the online focus group discussions to the experts via email. All the experts confirmed that the transcripts were a fair reflection of the focus group discussions. We employed thematic analysis by Braun and Clarke (2006) to analyse the 11 included articles and focus group discussions. Thematic analysis was used to provide a rich account of the data and to develop themes. As per the process of thematic analysis, we conducted substantive and axial coding of the raw data using the computer analysis software *NVivo 12* to reveal codes. Subsequently, we formulated categories, concepts and developed themes.

### Ethical considerations

This study was approved by the University of KwaZulu-Natal Biomedical Research Ethics Committee (BREC). Ethical clearance number: BF152/19.

## Results and discussion

In relation to the stated objective, to investigate the sensibility of food textural acoustics as a construct to understand eating for individuals with dysphagia, the results and discussion will be presented under the following two headings: (1) PRISMA-ScR results and bibliographic coverage findings of the 11 included articles, and (2) overview of core concepts and considerations related to food textural acoustics and eating.

### Preferred Reporting Items for Systematic reviews and Meta-analyses extension for Scoping Reviews (PRISMA-ScR) results and bibliographic coverage findings of the 11 included articles

There were 1439 articles identified in the initial search across the seven databases. After 741 duplicates were removed, both reviewers independently screened 228 articles. Appendix 3 depicts the results of the PRISMA-ScR and the bibliographic information of the 11 included articles. The results revealed that there were seven research studies and four narrative reviews. The studies were conducted over 2004–2017, with the majority of the studies (seven out of 11) conducted in the United Kingdom and the remaining studies conducted in Italy, Japan and Netherlands. Of the 11 articles, only one article was conducted in the field of speech and swallowing. Spence (field of experimental psychology) conducted a majority of studies (six out of 11) independently or with other researchers specifically from the fields of psychology. These findings depict that food textural acoustics has predominantly been researched in the field of psychology, denoting that in addition to the lack of diversity in geography, there is a lack of diversity in discipline and perspective exploring this construct.

### Overview of core concepts and considerations related to food textural acoustics and eating

To understand these results and discussion, it is essential to note that in addition to the thematic analysis, we analysed the revised scoping review and focus group discussions data using the critical theory and ideology critique framework. We used the interrogative framework by Guba and Lincoln (1994), adapted by Pillay (2003), and Pillay and Kathard (2018). This involved three assumptions that we used to formulate our three core arguments:

- Ontological assumption: Ontology is the nature of reality. Realities are subjective and it is dependent on each person (Parahoo, 2014). Realities are shaped by social, political, cultural and economic values (Guba & Lincoln, 1994).
- Epistemological assumption: Epistemology is truth bases. This refers to how knowledge is created and communicated (Denzin & Lincoln, 2017).
- Methodological assumption: This represents the research strategies used by researchers to confirm what they believe can be known (Denzin & Lincoln, 2017).

The analysis of the revised scoping review and the online focus group discussions revealed the following three interconnected themes, which we will discuss as three core arguments:

- The sensibility of the acoustic sense.
- It does the ‘boom’ on your palate.
- The measurement texture-lemma.

#### The sensibility of the acoustic sense

Eating involves multiple senses (Velasco, Carvalho, Petit, & Nijholt, 2016). However, acoustic properties are often overlooked and neglected in contributing to flavour perception (Spence, 2015). There were three references made in the included articles (A) (Article 5 (A5), A6 and A7) and four references during the focus group discussions regarding the neglect of the acoustic properties in comparison to the other senses. Participant 1 (P1), Hanson (a chemical engineer), referred to the auditory sense as the ‘hidden sense’. P4 (Zampini, a cognitive neuropsychologist) also described the acoustic sense as ‘one of the most neglected modalities when it comes to food perception’. This correlates with the literature that depicts that there is a lack of focus on food textural acoustics and the common reference to the auditory sense as the ‘forgotten flavour sense’ (Spence, 2015). There has been a recent renaissance of interest over the past 15–20 years in studying the acoustic sense in cognitive psychology, cognitive neuroscience, food science and gastronomical sciences (A5, A6, A7; Spence, Reinoso-Carvalho, Velasco, & Wang, 2019).

P4 further expressed that acoustic properties of food are rarely considered as influencing flavour perception. He believes that acoustics should be considered as the acoustic sense is a ‘relevant part of flavour experience’ and flavour involves more than smell and taste. Articles 2, 3 and 4 and article 9 made reference to auditory salience. When the acoustic properties of the food were modified and the texture remained unaltered, participants of these studies reported that the auditory information was more salient to them. This improved the participants’ ‘oral feel’ or oral somatosensation, perceiving the foods as harder or crispier (Spence & Zampini, 2006). This phenomenon was also depicted by a study by Masuda and Okajima (2011). These studies highlight the possible sensible nature of food textural acoustics in eating through the need to foreground the auditory sense. This should not be surprising as evolutionary, food acoustics has always served as a highly salient cue of freshness and pleasantness of food (A6; Tunick et al., 2013; Vickers, 1983). This notion of auditory salience is a critical factor to consider in the overall experience of eating for individuals with dysphagia.

#### It does the ‘boom’ on your palate

The analysis of the expert consultations and the 11 articles demonstrates six main codes that highlight the hedonic responses to food textural acoustics (i.e. enjoyment, positive feelings, intraoral sounds, food enhancement, freshness and texture perception). These references to the hedonic response to food textural acoustics were made mainly by P5 (Centelles, a sommelier). P5’s perspective predominantly focused on the need to build a dish that consists of auditory elements. He conveyed that this is needed as it ‘enhances’ the dish, ‘adds another dimension’, ‘flavours get more intense’ and is ‘something magical’ (Figure 2). The positive hedonic responses to food textural acoustics correlate with what is evident in the literature (Tunick et al., 2013; Vickers, 1983). These studies have reported that the characteristic of crispiness most strongly relates to a food’s pleasantness and enjoyment. A6 and Spence (2017) also depict that food acoustics improve the experience, pleasure and enjoyment of eating.

**FIGURE 2:**
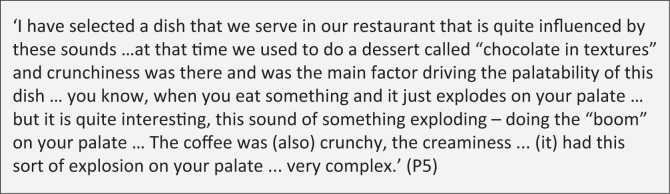
Box display of an excerpt from the consultation with participant 5 depicting hedonic response to food textural acoustics.

There are differing views and methods embraced to consider the hedonic influence of food acoustics. The analysis of the 11 articles and the vast literature shows that food textural acoustics has been focused on using a quasi-experimental orientation (A3, A4, A9 and A10; Masuda & Okajima, 2011). This involves subjectively evaluating hedonic responses related to the influence of food sounds on taste, freshness, feelings and texture perception using questionnaires (A3 and A4) and visual analogue scales (A1, A2 and A9). Conversely, P5 admitted that his perspectives are based purely on considering the feedback from his restaurant customers and their pleasure experiences. He further declared that this is ‘not scientific at all’. In the real world, cooks and chefs are known to be driven by this intuition and the need to foreground hedonic interests to food acoustics for consumers’ food experience, , as shown in Figure 2. This is also present in the creative dishes such as the *sound of the sea* by one of the world’s famous chefs Heston Blumenthal.

The above argument depicts that similar to cooks and chefs, experts affiliated with the scientific fields also express the significance of the hedonic responses to food textural acoustics. P3, Duizer (a food scientist), added that from a sensory perspective, sounds produced when eating indicates the freshness of food. Despite experts from the scientific fields expressing the importance of the hedonic responses, their focus is more closely aligned with the literature related to how the construct of food textural acoustics can be studied rather than solely based on intuition. Thus, a disparity exists between the varying perspectives of experts in the field, as well as the literature that portrays the reductionist nature adopted in the scientific fields. The reductionist approach does not admit into its purview sound because it is difficult to measure. There is also the opposing view that there is a need to focus on the body’s response and the hedonic value that sound has on the experience of eating. In relation to dysphagia management, in addition to considering the body’s response to food textural acoustics, it is essential to take into account premorbid food preferences and hedonic responses. Food palatability or positive hedonic response to foods are known to increase food intake, appetite and overall nutrition (A4; McCrickerd & Ford, 2016).

#### The measurement texture-lemma

Dysphagia practitioners view food and drink using a textural or rheological lens when recommending diet textural modifications. Traditionally in dysphagia science, dysphagia management has perpetuated a biomedical perspective. This perspective predominantly focuses on down modifications to make food safer for consumption to prevent choking and aspiration risks (i.e. moving from Level 6 or 7 to Level 3 or 4 based on the International Dysphagia Diet Standardisation Initiative framework) (Cichero et al., 2013; Cichero et al., 2017; IDDSI, 2019). This is possibly to prevent litigation issues arising from patient fatalities. Furthermore, the literature on dysphagia assessment and practice was mainly produced in the global north, promoting specific ontological viewpoints (nature of realities). This makes it difficult for the global south to adopt these viewpoints in practice as these contexts may be confounded by economic and social challenges such as reduced access to practitioner expertise and these assessment and management measures (Andrews & Pillay, 2017; Ostrofsky & Seedat, 2016).

We acknowledge that rheology is vital to foreground in diet textural modification as rheological properties of food contribute to swallowing. For instance, studies by Newman, Vilardell, Clavé and Speyer (2016) and Hadde, Cichero, Zhao Chen and Chen (2019) have depicted that rheological properties influence lingual pressure patterns, flow rate of the bolus, timing of the pharyngeal phase and swallow safety. Despite the documented benefits of diet textural modification, it should be noted that when texture or rheological properties of food are modified, the sensory and proprioceptive properties (i.e. visual, olfactory, tactile and acoustic properties) are also modified. This may lead to reduced enjoyment of the overall experience of eating. This makes it necessary to consider the holistic and complex nature of eating (i.e. textural and its associated acoustic properties), even in the management of individuals with dysphagia. This will be further elaborated on below.

**FIGURE 3:**
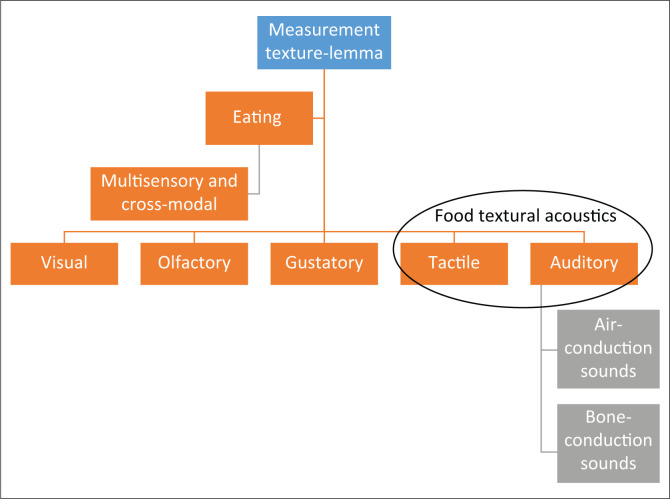
Diagram showing the complexity of eating and studying food textural acoustics.

**Multisensory and cross-modal nature of food:** Articles 2–11 and the expert consultations with P1, P4, P5 and P6 revealed two codes related to the complex nature of eating (i.e. multisensory and/or cross-modal integration) (Knöeferle & Spence, 2012). Argument one (sensibility of the acoustic sense) points to the sensibility of food textural acoustics as a construct to understand eating. Due to the complex nature of eating, it poses a methodological dilemma of measuring a specific sense like acoustics. P1, P4, P5 and P6 expressed that it will be challenging to study food acoustics as a single construct as ‘sound does not come alone…very tightly and close to texture and some other sensations…really complex topic’ (P5). P2, Wilson (an audiologist), also highlighted the intricacy of the processing of auditory stimuli. He described, ‘*It (auditory processing) is not linear or unidirectional*’, and it involves complex processes (i.e. various pattern matching and neural responses). The articles and the experts are in agreement with the literature that promotes the ontological appreciation of the notion of the complexity of eating as multisensory. A total of five articles (A2, A5, A6, A7 and A9) and two experts (P3 and P4) stated that acoustic properties of food have both air-conduction and bone-conduction influences, which further compounds this methodological dilemma (Christensen & Vickers, 1991; Dacremont, 1995; Dacremont & Colas, 1993; Duizer, 2001) (Figure 3).

Despite the acknowledgment of the complexity and interconnectedness of eating, empirical science and reductionist approaches have been adopted to study the auditory sense by isolating it (Endo, Ino, & Fujisaki, 2016, 2017; Masuda, Yamaguchi, Arai, & Okajima, 2008). This includes the use of data collection tools such as microphones with headphones (A2, A3, A4, A8, A9 and A10) and surface electrodes to detect mastication behaviours (A3 and A4). This reductionist science pulls out and manipulates the auditory cues in terms of its components (i.e. pitch, loudness and/or frequency and temporal factors) and it utilises such methods to gather information regarding the body’s responses. P6, Pillay (a speech pathologist and audiologist), expressed that isolating a sense is ‘artificial and sanitised that it actually takes away from the natural event (of eating) itself’. He further reported that using this type of approach becomes less pragmatic and less implementable in our work as practitioners. By virtue of the fact that eating is both multisensory and cross-modal, eating needs to be understood holistically. Studying eating by isolating the various senses may be of value (such as those conducted by the studies described above); however, it is counteractive. We need to acknowledge this blind spot and rethink this paradigm when studying the construct. P6 voiced that he believes that the difficulty in measuring the construct ‘comes from the methodology we use, the science we use’. This will be discussed further below.

**The soundness of positioning:** The results of the expert consultations exposed three primary codes related to positioning of food textural acoustics (i.e. bias, variety of perspectives and knowledge fields, and the culture of the science that we follow). Varying perspectives emerged from the expert consultations and this illuminated the notion of memetics. Memetics theoretically originates from the central concept of a meme (unit of cultural evolution and selection), conceptualised by Richard Dawkins in 1977 (Tyler, 2011). We have operationalised the notion of memetics in this study as the way in which ideas have been generated across time and space (Castaño Díaz, 2013; Dawkins, 1977). P4 stated that he felt ‘comfortable’ with the science that he uses. P2 and P6 had an overt discussion of the science. P2 indicated at the onset that his thoughts reflect his biases of understanding auditory processing from more of a bottom-up perspective (‘that goes from outer ear to middle ear to inner ear to cochlear nerve, to cochlear nucleus… station by station up the pathway’). This was because of his profession and retreat to topics that he is more comfortable with. This bottom-up perspective correlates with A5 and A7 which reviews highly controlled laboratory-based studies, and the literature that acknowledges more epistemological variations that come from quasi-experimental or positivistic frameworks (Endo et al., 2016, 2017; Masuda et al., 2008). Despite P2’s stated professional bias, he questions certain scientific basis and encourages that auditory processing be understood from a more top-down perspective. This includes consideration of the influence of higher-order functions such as expectation and memory (Spence, 2015; Piqueras-Fiszman, 2020; Sakai, 2020). Similarly, we need to consider these higher-order functions during eating and also for those with dysphagia (i.e. premorbid food preferences, and anticipation for consumption and subsequent memories of food experiences) (A6).

It is crucial to acknowledge the methodological dilemma (i.e. the complexity of eating because of its multisensory and cross-modal nature) and approach used when studying the construct of food textural acoustics. This may assist in conceptualising and developing innovative methods of studying the construct. Isolating a sense may not be the most ideal approach. P6, a South African–based practitioner proposed, ‘our mission is to look at inventing or imagining a new science that can help us take this (construct) forward’. The multisensory nature of eating with the consideration of the textural, and its associated acoustic, properties should be foregrounded in the assessment and management of individuals with dysphagia. P6 further expressed:

‘We work in a low socio-economic context. And a lot of the people we work with (individuals with dysphagia) do not have enough healthcare services to help… So we want to invest in developing foods that are in and of themselves maximally therapeutic… for its impact on the swallow mechanism. If sound is one of the other ingredients we can put into it (dysphagia diets), then that is why we want to develop it.’ (P6, Pillay, Speech Pathologist and Audiologist )

### ‘Illuminators’ and implications for the field of dysphagia

The above three intersecting arguments illustrate that the construct of food textural acoustics may be sensible to investigate as it is supported from both a practice and theoretical perspective and from the focus of empirical-based and ecological studies. Why have we not sufficiently researched and factored food textural acoustics and its potential therapeutic and hedonic benefits into dysphagia practice? We are more focused on diet textural modifications such as recommending softened foods and the use of commercial food thickeners. This often results in undernutrition, reduced quality of life, poor compliance and frequent hospitalisations because of recurrent aspiration pneumonia (O’Keeffe, 2018; Shune & Namasivayam-MacDonald, 2020; Swan et al., 2015). Are we solely concerned with food safety, rather than with rehabilitating swallowing and improving overall quality of life? Are we treating the condition of dysphagia instead of the whole individual?

The research across the fields of psychology, cognitive neuroscience, gastronomical sciences and marketing has depicted the influence of food acoustics on behaviour and perception (Callan et al., 2018; Carvalho et al., 2016; Höchenberger & Ohla, 2019; Kantono et al., 2018; Spence, 2015, 2017). How can we understand the influence of food textural acoustics on the body’s physiological response for dysphagia management? A few illuminators exist to connect this construct of food textural acoustics to dysphagia. However, two will be discussed below.

Studies by Endo et al. (2016, 2017) have investigated the influence of altered auditory feedback of chewing sounds, which resembled a crunchy sound on the perception of food texture. The results revealed that in addition to positively influencing palatability and taste, this pseudo-chewing sound had an influence on the perception of food texture (i.e. perceiving the texture as stiff and rougher). This influence was present even in the absence of the actual crunchy oral sensation. This technique may be used to improve the texture perception and hedonic responses to texturally modified foods for individuals with dysphagia, without changing the actual texture of the foods. A second illuminator is the possible therapeutic benefit of ‘up-modifications’ (i.e. moving from lower Level 4 to Level 6 on the IDDSI framework) (refer to Pillay [2013] for further explanation). This involves the use of transitional foods for individuals with dysphagia. Transitional foods (also known as solid meltable foods) refer to foods that change rapidly by melting when it comes into contact with body temperature, becoming easier to chew and swallow (Cichero et al., 2017). An example in the South African context is the Bakers Blue Label® Marie Biscuit. The most recent version of the IDDSI framework includes transitional foods that span across the pyramid. Research has shown the benefit of transitional foods in the paediatric populations, especially for those with less mature or underdeveloped sensorimotor systems for mastication (Dovey, Aldridge, & Martin, 2013; Gisel, 1991). This offers some promise for the adult population. A study by Barewal, Shune, Ball and Kosty (2020) demonstrated that it is necessary to consider the use of transitional foods like *Savorease* and the *EAT-Bar,* which are two commercially available transitional foods. These transitional foods may provide improved eating pleasure and nutrition for individuals on texturally modified foods (Barewal et al., 2020). Snack foods and finger foods like the *Savorease* require minimal chewing that may benefit masticatory muscle strength and cognition for individuals with dysphagia (Barewal et al., 2020). These studies described above provide a strong evidence to support the development of more ‘sensory-responsive foods’, as per the THRIVE programme, because of their improved pleasure and potential therapeutic benefit.

### Study critique

We used multiple data sources (i.e. revised scoping review, online consultation phase and reference to published data) and data analysis methods. This was used as a means of triangulation to improve the credibility and trustworthiness of the study. The inclusion of a pilot study and a blind review prior to and during the scoping review aided with establishing data credibility. We acknowledge the limitations associated with the scoping review methodology. For instance, the search terms and databases chosen might have resulted in some relevant articles being overlooked. However, we used a rigorous method for data collection to alleviate this potential limitation. The co-author of this study acted as a supervisor and as an informant during the expert consultations. While this may appear to be bias, he was included as this study followed a hermeneutic paradigm (Guba & Lincoln, 1994). This involved including individuals’ opinions as a way of acknowledging that multiple realities exist and foregrounding and celebrating subjectivities (Guba & Lincoln, 1994; Pillay & Kathard, 2018). Following the expert consultations, we also conducted member checking to promote trustworthy and credible data (Birt et al., 2016; Cope, 2014).

## Conclusion

This study has synthesised the literature, especially from 1980 to 2020, and views from world experts from various fields. The results of this study depict the possible sensibility of food textural acoustics as a construct to understand eating for individuals with dysphagia. We have highlighted auditory salience numerous times throughout the article with the need to change the rhetoric of sound the ‘forgotten flavour sense’ (Spence, 2015) to the ‘celebrated flavour sense’. Thoughts influence practice. We hope that this article inspires researchers and practitioners to think differently by using texture, and its associated acoustic, properties such as the use of transitional foods as a way to reimagine dysphagia practice. This study may drive us to develop novel ways of approaching this construct and to explore its potential clinical applicability for individuals with dysphagia. This may be particularly contextually responsive to those individuals with dysphagia from low- to middle-income contexts such as South Africa and Brazil, where food security and food sovereignty are a concern.

## Figures and Tables

**FIGURE 1-A2:**
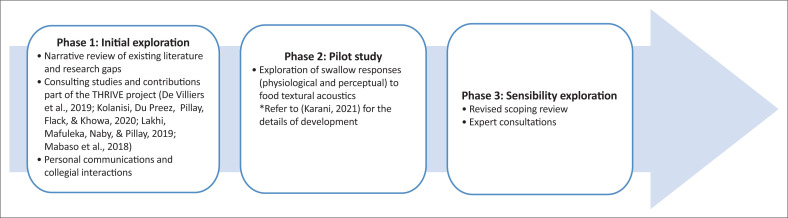
The broader study involved three main phases (initial exploration, pilot study and sensibility exploration). Phase 3 formed the primary focus of this article.

**FIGURE 2-A2:**
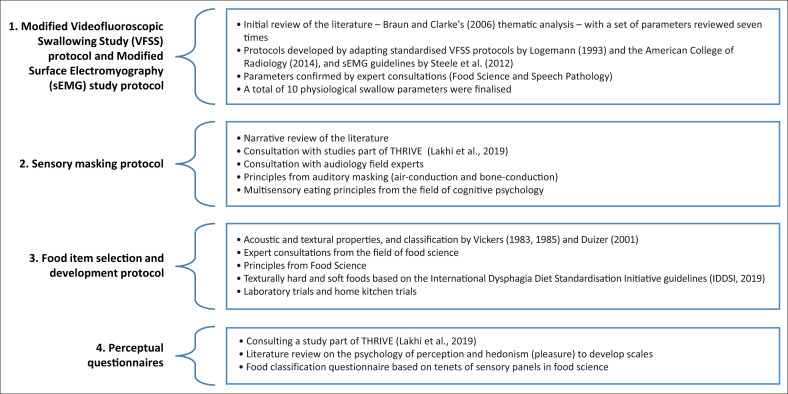
Self-developed protocols and tools associated with phase 2.

**FIGURE 1-A3:**
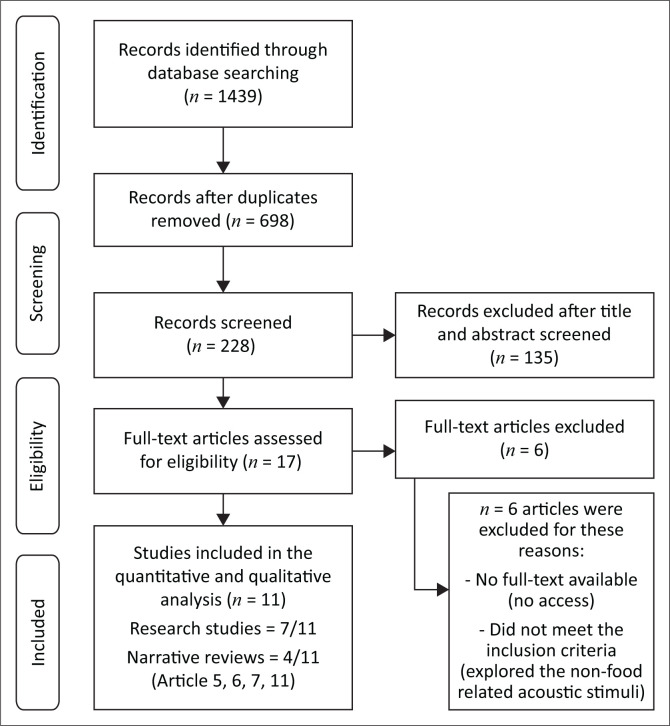
Preferred Reporting Items for Systematic Reviews and Meta-analyses extension for Scoping Review (PRISMA-ScR) flow diagram results.

## Appendix 1

**TABLE 1-A1:** Established fields that have alluded to food textural acoustics.

Fields	Reference
**Engineering and physical sciences (Chemistry)**	Acoustic Rheology: (Peleg, 2017; Zadeike et al., 2018)
**Food science**	Drake (1965) was the pioneer in investigating and noting the relationship between the auditory sense in texture perception and described this as textural acoustics. Duizer (2001): Food acoustic classification system (crispy, crunchy and crackly)
**Gastronomical sciences**	Food marketing and packaging (Spence, 2016; Wang & Spence, 2019)
**Cognitive neurosciences**	Music: (Carvalho et al., 2016; Kantono et al., 2016; Kantano et al., 2018) Background sounds: (Callan, Callan, & Ando, 2018; Lowe, Rigler, & Haws, 2018; Rahne, Köppke, Nehring, Plontke, & Fischer, 2018; Trautmann, Meier- Dinkel, Gertheiss, & Mörlein, 2017)
**Dysphagia sciences**	Textural modifications: (Cichero, 2018; Cichero et al., 2017; De Villiers et al., 2019; IDDSI, 2019; Swan et al., 2015)

## Appendix 4

**TABLE 1-A4:** Bibliographic data of the 11 included articles.

Article number	Title	Author/s	Year	Location in which the study was conducted	Knowledge field	Affiliation (Journal)
1	Does listening to the sound of yourself chewing increase your enjoyment of food?	Amos et al.	2006	United Kingdom	Speech and Swallowing Research	*Annals of General Psychiatry*
2	Effects of the sound of the bite on apple perceived crispness and hardness.	Demattè et al.	2014	Italy	Psychology and Cognitive Science	*Food Quality and Preference*
3	The effect of a crunchy pseudo-chewing sound on perceived texture of softened foods.	Endo et al.	2016	Japan	Industrial Science and Technology	*Physiology & Behavior*
4	Texture-dependent effects of pseudo-chewing sound on perceived food texture and evoked feelings in response to nursing care foods.	Endo et al.	2017	Japan	Industrial Science and Technology	*Appetite*
5	Auditory contributions to flavour perception and feeding behaviour.	Spence	2012	United Kingdom	Experimental Psychology	*Physiology & Behavior*
6	Eating with our ears: assessing the importance of the sounds of consumption on our perception and enjoyment of multisensory flavour experiences.	Spence	2015	United Kingdom	Experimental Psychology	*Flavour*
7	The influence of auditory cues on the perception of, and responses to, food and drink.	Spence and Shankar	2010	United Kingdom	Experimental Psychology	*Journal of Sensory Studies*
8	The influence of auditory and visual information on the neuromuscular control of chewing crispy food.	Van der Bilt, Pocztaruk, Frasca, Van der Glas, and Abbink	2011	Netherlands Brazil	Oral-Maxillofacial Surgery, Prosthodontics and Special Dental Care Faculty of Dentistry	*European Journal of Oral Sciences*
9	The role of auditory cues in modulating the perceived crispness and staleness of potato chips.	Zampini and Spence	2004	United Kingdom	Experimental Psychology	*Journal of Sensory Studies*
10	Modifying the multisensory perception of a carbonated beverage using auditory cues.	Zampini and Spence	2005	United Kingdom	Experimental Psychology	*Food Quality and Preference*
11	Assessing the role of sound in the perception of food and drink.	Zampini and Spence	2010	United Kingdom Italy	Experimental Psychology Cognitive Sciences	*Chemosensory Perception*

## References

[CIT0001] Alvesson, M., & Sköldberg, K. (2009). *Reflexive methodology: New vistas for qualitative research* (2nd edn.). London: Sage.

[CIT0002] American College of Radiology. (2014). *ACR practice parameter for the performance of the Modified Barium Swallow*. Retrieved from http://www.acr.org/~/media/ACR/Documents/PGTS/guidelines/Modified_Barium_Swallow.pdf

[CIT0003] Amos, K.E., Anari, S., Buswell, C.A., McNeill, E.J., Printza, A., Ray, S.J., & Rustom, I. (2006). Does listening to the sound of yourself chewing increase your enjoyment of food? *Annals of General Psychiatry*, 5(1), 22. 10.1186/1744-859X-5-22 17134513 PMC1698474

[CIT0004] Andrews, M., & Pillay, M. (2017). Poor consistency in evaluating South African adults with neurogenic dysphagia. *South African Journal of Communication Disorders*, 64(1), 1–14. 10.4102/sajcd.v64i1.158 PMC5842977 28155280

[CIT0005] Arksey, H., & O’Malley, L. (2005). Scoping studies: towards a methodological framework. *International Journal of Social Research Methodology*, 8(1), 19–32.

[CIT0006] Barewal, R., Shune, S., Ball, J., & Kosty, D. (2020). A comparison of behavior of transitionalstate foods under varying oral conditions. *Dysphagia*, 36, 316–324. 10.1007/s00455-020-10135-w 32458146

[CIT0007] Birt, L., Scott, S., Cavers, D., Campbell, C., & Walter, F. (2016). Member checking: A tool to enhance trustworthiness or merely a nod to validation? *Qualitative Health Research*, 26(13), 1802–1811. 10.1177/1049732316654870 27340178

[CIT0008] Braun, V., & Clarke, V. (2006). Using thematic analysis in psychology. *Qualitative Research in Psychology*, 3(2), 77–101. 10.1191/1478088706qp063oa

[CIT0009] Callan, A., Callan, D., & Ando, H. (2018, 6 14–17). Differential effects of music and pictures on taste perception – an fMRI study [Poster presentation]. Toronto, CA: Annual meeting of the International Multisensory Research Forum.

[CIT0010] Carvalho, F.R., Wang, Q.J., Van Ee, R., & Spence, C. (2016). The influence of soundscapes on the perception and evaluation of beers. *Food Quality and Preference*, 52, 32–41. 10.1016/j.foodqual.2016.03.009

[CIT0011] Castaño Díaz, C.M. (2013). Defining and characterizing the concept of Internet Meme. *Ces Psicología*, 6(2), 82–104.

[CIT0012] Christensen, C.M., & Vickers, Z.M. (1981). Relationships of chewing sounds to judgments of food crispness. *Journal of Food Science*, 46(2), 574–578. 10.1111/j.1365-2621.1981.tb04914.x

[CIT0013] Cichero, J.A. (2018). Age-related changes to eating and swallowing impact frailty: Aspiration, choking risk, modified food texture and autonomy of choice. *Geriatrics*, 3(4), 69. 10.3390/geriatrics3040069 PMC6371116 31011104

[CIT0014] Cichero, J.A., Lam, P., Steele, C.M., Hanson, B., Chen, J., Dantas, R.O., … & Pillay, M. (2017). Development of international terminology and definitions for texture-modified foods and thickened fluids used in dysphagia management: The IDDSI framework. *Dysphagia*, 32(2), 293–314. 10.1007/s00455-016-9758-y 27913916 PMC5380696

[CIT0015] Cichero, J.A., Steele, C., Duivestein, J., Clavé, P., Chen, J., Kayashita, J., … & Murray, J. (2013). The need for international terminology and definitions for texture-modified foods and thickened liquids used in dysphagia management: Foundations of a global initiative. *Current Physical Medicine and Rehabilitation Reports*, 1(4), 280–291. 10.1007/s40141-013-0024-z 24392282 PMC3873065

[CIT0016] Colquhoun, H.L., Jesus, T.S., O’Brien, K.K., Tricco, A.C., Chui, A., Zarin, W., … Straus, S. (2017). Study protocol for a scoping review on rehabilitation scoping reviews. *Clinical Rehabilitation*, 31(9), 1249–1256. 28118743 10.1177/0269215516688514

[CIT0017] Cope, D.G. (2014). Methods and meanings: Credibility and trustworthiness of qualitative research. *Oncology Nursing Forum*, 41(1), 89–91. 24368242 10.1188/14.ONF.89-91

[CIT0018] Coppin, G. (2020). The COVID-19 may help enlightening how emotional food is. *NPJ Science of Food*, 4(1), 1–3. 10.1038/s41538-020-00071-2 32821852 PMC7423882

[CIT0019] Dacremont, C. (1995). Spectral composition of eating sounds generated by crispy, crunchy and crackly foods. *Journal of Texture Studies*, 26(1), 27–43. 10.1111/j.1745-4603.1995.tb00782.x

[CIT0020] Dacremont, C., & Colas, B. (1993). Effect of visual clues on evaluation of bite sounds of foodstuffs. *Sciences des Aliments*, 13(4), 603–610.

[CIT0021] Dawkins, R. (1977). The selfish gene (1989 edn.). Oxford: Oxford University Press.

[CIT0022] De Villiers, M., Hanson, B., Moodley, L., & Pillay, M. (2019). The impact of modification techniques on the rheological properties of dysphagia foods and liquids. *Journal of Texture Studies*, 51(1), 154–168. 10.1111/jtxs.12476 31397895

[CIT0023] Demattè, M.L., Pojer, N., Endrizzi, I., Corollaro, M.L., Betta, E., Aprea, E., … Gasperi, F. (2014). Effects of the sound of the bite on apple perceived crispness and hardness. *Food Quality and Preference*, 38, 58–64. 10.1016/j.foodqual.2014.05.009

[CIT0024] Denzin, N.K., & Lincoln, Y.S. (Eds.). (2017). *The Sage handbook of qualitative research*. London: Sage.

[CIT0025] Dodd, B. (2007). Evidence-based practice and speech-language pathology: Strengths, weaknesses, opportunities and threats. *Folia Phoniatrica et logopaedica*, 59(3), 118–129. 10.1159/000101770 17556855

[CIT0026] Dovey, T.M., Aldridge, V.K., & Martin, C.I. (2013). Measuring oral sensitivity in clinical practice: A quick and reliable behavioural method. *Dysphagia*, 28(4), 501–510. 10.1007/s00455-013-9460-2 23515637

[CIT0027] Drake, B.K. (1965). Food crushing sounds: Comparisons of objective and subjective data. *Journal of Food Science*, 30(3), 556–559. 10.1111/j.1365-2621.1965.tb01801.x

[CIT0028] Duizer, L. (2001). A review of acoustic research for studying the sensory perception of crisp, crunchy and crackly textures. *Trends in Food Science & Technology*, 12(1), 17–24. 10.1016/S0924-2244(01)00050-4

[CIT0029] Endo, H., Ino, S., & Fujisaki, W. (2016). The effect of a crunchy pseudo-chewing sound on perceived texture of softened foods. *Physiology & Behavior*, 167, 324–331. 10.1016/j.physbeh.2016.10.001 27720736

[CIT0030] Endo, H., Ino, S., & Fujisaki, W. (2017). Texture-dependent effects of pseudo-chewing sound on perceived food texture and evoked feelings in response to nursing care foods. *Appetite*, 116, 493–550. 10.1016/j.appet.2017.05.051 28572067

[CIT0031] Gisel, E.G. (1991). Effect of food texture on the development of chewing of children between six months and two years of age. *Developmental Medicine & Child Neurology*, 33(1), 69–79. 10.1111/j.1469-8749.1991.tb14786.x 1995410

[CIT0032] Guba, E.G., & Lincoln, Y.S. (1994). Competing paradigms in qualitative research. *Handbook of Qualitative Research*, 2(163–194), 105.

[CIT0033] Hadde, E.K., Cichero, J.A.Y., Zhao, S., Chen, W., & Chen, J. (2019). The importance of extensional rheology in bolus control during swallowing. *Scientific Reports*, 9(1), 1–10. 10.1038/s41598-019-52269-4 31695062 PMC6834566

[CIT0034] Harris, A. (2015). *Autophony: Listening to your eyes move*. Somatosphere: Science, Medicine and Anthropology. Retrieved October 18, 2020 from http://somatosphere.net/2015/06/autophony-listening-to-your-eyes-move.html

[CIT0035] Höchenberger, R., & Ohla, K. (2019). A bittersweet symphony: Evidence for taste-sound correspondences without effects on taste quality-specific perception. *Journal of Neuroscience Research*, 97(3), 267–275. 10.1002/jnr.24308 30027567

[CIT0036] In, J. (2017). Introduction of a pilot study. *Korean Journal of Anesthesiology*, 70(6), 601. 10.4097/kjae.2017.70.6.601 29225742 PMC5716817

[CIT0037] International Diet Standardisation Initiative. (2019). *IDDSI framework and detailed level definitions*. Retrieved November 10, 2020 from https://ftp.iddsi.org/Documents/Complete_IDDSI_Framework_Final_31July2019.pdf

[CIT0038] Kantono, K., Hamid, N., Shepherd, D., Lin, Y.H.T., Brard, C., Grazioli, G., & Carr, B.T. (2018). The effect of music on gelato perception in different eating contexts. *Food Research International*, 113, 43–56. 10.1016/j.foodres.2018.06.030 30195538

[CIT0039] Kantono, K., Hamid, N., Shepherd, D., Yoo, M.J., Grazioli, G., & Carr, B.T. (2016). Listening to music can influence hedonic and sensory perceptions of gelati. *Appetite*, 100, 244–255. 10.1016/j.appet.2016.02.143 26923742

[CIT0040] Karani, T.F. (2021). *Crispy, crunchy and crackly: An exploration of food textural acoustics on the swallow mechanism*. Unpublished PhD dissertation. University of KwaZulu-Natal, South Africa.

[CIT0041] Knöferle, K., & Spence, C. (2012). Crossmodal correspondences between sounds and tastes. *Psychonomic Bulletin & Review*, (19), 992–1006. 10.3758/s13423-012-0321-z 23055144

[CIT0042] Lakhi, L., Mafuleka, A., Naby, S., & Pillay. (2019). *An exploration of the physiological swallowing responses to the acoustic properties of texturally modified foods*. Unpublished undergraduate dissertation. University of KwaZulu-Natal.

[CIT0043] Levac, D., Colquhoun, H., & O’Brien, K.K. (2010). Scoping studies: Advancing the methodology. *Implementation Science*, 5(1), 69. 10.1186/1748-5908-5-69 20854677 PMC2954944

[CIT0044] Little, L.M., Dean, E., Tomchek, S.D., & Dunn, W. (2017). Classifying sensory profiles of children in the general population. *Child: Care, Health and Development*, 43(1), 81–88. 10.1111/cch.12391 27545764

[CIT0045] Logemann, J.A. (1993). *Manual for the videofluorographic study of swallowing*. Austin, TX: Pro-Ed.

[CIT0046] Lowe, M., Ringler, C., & Haws, K. (2018). An overture to overeating: The cross-modal effects of acoustic pitch on food preferences and serving behaviour. *Appetite*, 123, 128–134. 10.1016/j.appet.2017.12.013 29253670

[CIT0047] Mabaso, M., Malinga, T., & Paruk, J. (2018). *The establishment of normative data on healthy young adults for complex auditory brainstem response testing at University of KwaZulu-Natal, Audiology clinic*. Unpublished undergraduate dissertation. University of KwaZulu-Natal.

[CIT0048] Masuda, M., & Okajima, K. (2011). Added mastication sound affects food texture and pleasantness. *i-Perception*, 2(8), 949–949. 10.1068/ic949

[CIT0049] Masuda, M., Yamaguchi, Y., Arai, K., & Okajima, K. (2008). Effect of auditory information on food recognition. *IEICE Technical Report*, 108, 123–126.

[CIT0050] McCrickerd, K., & Forde, C.G. (2016). Sensory influences on food intake control: moving beyond palatability. *Obesity Reviews*, 17(1), 18–29. 26662879 10.1111/obr.12340

[CIT0051] Menni, C., Valdes, A.M., Freidin, M.B., Sudre, C.H., Nguyen, L.H., Drew, D.A., … Spector, T.D. (2020). Real-time tracking of self-reported symptoms to predict potential COVID-19. *Nature Medicine*, 26(7), 1037–1040. 10.1038/s41591-020-0916-2 PMC7751267 32393804

[CIT0052] Moola, S., Munn, Z., Sears, K., Sfetcu, R., Currie, M., Lisy, K., … Mu, P. (2015). Conducting systematic reviews of association (aetiology): The Joanna Briggs Institute’s approach. *International Journal of Evidence-Based Healthcare*, 13(3), 163–169. 10.1097/XEB.0000000000000064 26262566

[CIT0053] Newman, R., Vilardell, N., Clavé, P., & Speyer, R. (2016). Effect of bolus viscosity on the safety and efficacy of swallowing and the kinematics of the swallow response in patients with oropharyngeal dysphagia: White paper by the European Society for Swallowing Disorders (ESSD). *Dysphagia*, 31(2), 232–249. 10.1007/s00455-016-9696-8 27016216 PMC4929168

[CIT0054] O’Keeffe, S.T. (2018). Use of modified diets to prevent aspiration in oropharyngeal dysphagia: Is current practice justified? *BMC Geriatrics*, 18(1), 1–10. 10.1186/s12877-018-0839-7 30029632 PMC6053717

[CIT0055] Ostrofsky, C., & Seedat, J. (2016). The South African dysphagia screening tool (SADS): A screening tool for a developing context. *South African Journal of Communication Disorders*, 63(1), 1–9. 10.4102/sajcd.v63i1.117 PMC5843204 26974244

[CIT0056] Palumbo, D.B., Alsalman, O., De Ridder, D., Song, J.J., & Vanneste, S. (2018). Misophonia and potential underlying mechanisms: A perspective. *Frontiers in Psychology*, 9, 953. 10.3389/fpsyg.2018.00953 30008683 PMC6034066

[CIT0057] Parahoo, K. (2014). *Nursing research: Principles, process and issues* (3rd ed.). Basingstoke: Palgrave Macmillan International Higher Education.

[CIT0058] Parma, V., Ohla, K., Veldhuizen, M.G., Niv, M.Y., Kelly, C.E., Bakke, A.J., … Kaur, R. (2020). More than smell – COVID-19 is associated with severe impairment of smell, taste, and chemesthesis. *Chemical Senses*, 45(7), 609–622. 10.1093/chemse/bjaa041 32564071 PMC7337664

[CIT0059] Peleg, M. (2017). The basics of solid foods rheology. In Moskowitz (eds.). *Food texture* (pp. 3–33). Boca Raton: Routledge.

[CIT0060] Pillay, M. (2003). Cross-cultural practice: What is it really about? *Folia phoniatrica et logopaedica*, 55(6), 293–299. 10.1159/000073252 14573985

[CIT0061] Pillay, M. (2013, 8 25–29). Should poor people, who cannot eat or drink safely, be treated differently?: Dysphagia services in resource constrained contexts [Panel presentation]. Turin: International Association for Logopedics & Phoniatrics.

[CIT0062] Pillay, M., & Kathard, H. (2018). Renewing our cultural borderlands. *Topics in Language Disorders*, 38(2), 143–160. 10.1097/TLD.0000000000000151

[CIT0063] Piqueras-Fiszman, B. (2020). The Psychology of Food Choice: Anticipation and Mental Simulation. *Handbook of Eating and Drinking: Interdisciplinary Perspectives*, 185–198.

[CIT0064] Rahne, T., Köppke, R., Nehring, M., Plontke, S.K., & Fischer, H.-G. (2018). Does ambient noise or hypobaric atmosphere influence olfactory and gustatory function? *PLoS One*, 13(1), e0190837. 10.1371/journal.pone.0190837 29370217 PMC5784903

[CIT0065] Sackett, D.L., & Rosenberg, W.M.C. (1995). On the need for evidence-based medicine. *Journal of Public Health*, 17(3), 330–334. 8527187

[CIT0066] Sakai, N. (2020). Top-down processing in food perception: Beyond the multisensory processing. *Acoustical Science and Technology*, 41(1), 182–188.

[CIT0067] Schultz, A., Goertzen, L., Rothney, J., Wener, P., Enns, J., Halas, G., & Katz, A. (2018). A scoping approach to systematically review published reviews: Adaptations and recommendations. *Research Synthesis Methods*, 9(1), 116–123. 10.1002/jrsm.1272 29032590

[CIT0068] Shune, S.E., & Namasivayam-MacDonald, A. (2020). Dysphagia-related caregiver burden: Moving beyond the physiological impairment. *Perspectives of the ASHA Special Interest Groups*, 5(5), 1282–1289. 10.1044/2020_PERSP-20-00067

[CIT0069] Spence, C. (2015). Multisensory flavor perception. *Cell*, 161(1), 24–35. 10.1016/j.cell.2015.03.007 25815982

[CIT0070] Spence, C. (2016). Multisensory packaging design: Color, shape, texture, sound, and smell. In Peter Burgess (Eds.), *Integrating the packaging and product experience in food and beverages* (pp. 1–22). Cambridge: Woodhead Publishing.

[CIT0071] Spence, C. (2017). *Gastrophysics: The new science of eating*. Viking, New York.

[CIT0072] Spence, C. (2020). Multisensory flavour perception: Blending, mixing, fusion, and pairing within and between the senses. *Foods*, 9(4), 407. 10.3390/foods9040407 PMC7230593 32244690

[CIT0073] Spence, C., & Shankar, M.U. (2010). The influence of auditory cues on the perception of, and responses to, food and drink. *Journal of Sensory Studies*, 25(3), 406–430. 10.1111/j.1745-459X.2009.00267.x

[CIT0074] Spence, C., Reinoso-Carvalho, F., Velasco, C., & Wang, Q.J. (2019). Extrinsic auditory contributions to food perception & consumer behaviour: An interdisciplinary review. *Multisensory Research*, 32(4–5), 275–318. 10.1163/22134808-20191403 31059484

[CIT0075] Spence, C., & Zampini, M. (2006). Auditory contributions to multisensory product perception. *Acta Acustica united with Acustica*, 92(6), 1009–1025.

[CIT0076] Steele, C.M., Alsanei, W.A., Ayanikalath, S., Barbon, C.E., Chen, J., Cichero, J.A., … Hanson, B. (2015). The influence of food texture and liquid consistency modification on swallowing physiology and function: a systematic review. *Dysphagia*, 30(1), 2–26. 10.1007/s00455-014-9578-x 25343878 PMC4342510

[CIT0077] Steele, C.M., Bennett, J.W., Chapman-Jay, S., Cliffe Polacco, R., Molfenter, S.M., & Oshalla, M. (2012). Electromyography as a biofeedback tool for rehabilitating swallowing muscle function. *Applications of EMG in Clinical and Sports Medicine*, InTech; 311–328.

[CIT0078] Steele, C.M., & Miller, A.J. (2010). Sensory input pathways and mechanisms in swallowing: A review. *Dysphagia*, 25(4), 323–333. 10.1007/s00455-010-9301-5 20814803 PMC2992653

[CIT0079] Strati, A. (2007). Sensible knowledge and practice-based learning. *Management Learning*, 38(1), 61–77. 10.1177/1350507607073023

[CIT0080] Swan, K., Speyer, R., Heijnen, B.J., Wagg, B., & Cordier, R. (2015). Living with oropharyngeal dysphagia: Effects of bolus modification on health-related quality of life – A systematic review. *Quality of Life Research*, 24(10), 2447–2456. 10.1007/s11136-015-0990-y 25869989

[CIT0081] Tidball, G.A., & Fagelson, M. (2018). Audiological assessment of decreased sound tolerance. In M. Fagelson and D.M Baguley (Eds.), *Hyperacusis and disorders of sound intolerance* (pp. 15–32). San Diego, CA: Plural Publishing.

[CIT0082] Trautmann, J., Meier-Dinkel, L., Gertheiss, J., & Mörlein, D. (2017). Noise and accustomation: A pilot study of trained assessors’ olfactory performance. *PLoS One*, 12(4), e0183875. 10.1371/journal.pone.0174697 28380041 PMC5381871

[CIT0083] Tricco, A.C., Lillie, E., Zarin, W., O’Brien, K.K., Colquhoun, H., Levac, D., … Straus, S.E. (2018). PRISMA extension for scoping reviews (PRISMA-ScR): Checklist and explanation. *Annals of Internal Medicine*, 169(7), 467–473. 30178033 10.7326/M18-0850

[CIT0084] Tunick, M.H., Onwulata, C.I., Thomas, A.E., Phillips, J.G., Mukhopadhyay, S., Sheen, S., … Cooke, P.H. (2013). Critical evaluation of crispy and crunchy textures: A review. *International Journal of Food Properties*, 16(5), 949–963. 10.1080/10942912.2011.573116

[CIT0085] Tyler, T. (2011). *Memetics: Memes and the science of cultural evolution*. Grenoble: Mersenne Publishing.

[CIT0086] Van der Bilt, A., Pocztaruk, R.L., Frasca, L.C., Van der Glas, H.W., & Abbink, J.H. (2011). The influence of auditory and visual information on the neuromuscular control of chewing crispy food. *European Journal of Oral Sciences*, 119(6), 427–434. 10.1111/j.1600-0722.2011.00878.x 22112027

[CIT0087] Velasco, C., Carvalho, F.R., Petit, O., & Nijholt, A. (2016, 11 16). A multisensory approach for the design of food and drink enhancing sonic systems. In *Proceedings of the 1st workshop on multi-sensorial approaches to human-food interaction* (pp. 1–7). New York: Association for Computing Machinery.

[CIT0088] Verhagen, J.V. (2007). The neurocognitive bases of human multimodal food perception: consciousness. *Brain Research Reviews*, 53(2), 271–286. 10.1016/j.brainresrev.2006.09.002 17027988 PMC3373180

[CIT0089] Vickers, Z.M. (1983). Pleasantness of food sounds. *Journal of Food Science*, 48(3), 783–786. 10.1111/j.1365-2621.1983.tb14898.x

[CIT0090] Vickers, Z.M. (1985). The relationships of pitch, loudness and eating technique to judgments of the crispness and crunchiness of food sounds 2. *Journal of Texture Studies*, 16(1), 85–95. 10.1111/j.1745-4603.1985.tb00681.x

[CIT0091] Vickers, Z.A.T.A., & Bourne, M.C. (1976). A psychoacoustical theory of crispness. *Journal of Food Science*, 41(5), 1158–1164. 10.1111/j.1365-2621.1976.tb14407.x

[CIT0092] Wang, Q.J., & Spence, C. (2019). Sonic packaging: how packaging sounds influence multisensory product evaluation. In C. Velasco & C. Spence (Eds.), *Multisensory packaging* (pp. 103–125). Cham: Palgrave Macmillan.

[CIT0093] Zadeike, D., Jukonyte, R., Juodeikiene, G., Bartkiene, E., & Valatkeviciene, Z. (2018). Comparative study of ciabatta crust crispness through acoustic and mechanical methods: Effects of wheat malt and protease on dough rheology and crust crispness retention during storage. *LWT*, 89, 110–116. 10.1016/j.lwt.2017.10.034

[CIT0094] Zampini, M., & Spence, C. (2004). The role of auditory cues in modulating the perceived crispness and staleness of potato chips. *Journal of Sensory Studies*, 19(5), 347–363. 10.1111/j.1745-459x.2004.080403.x

[CIT0095] Zampini, M., & Spence, C. (2005). Modifying the multisensory perception of a carbonated beverage using auditory cues. *Food Quality and Preference*, 16(7), 632–641. 10.1016/j.foodqual.2004.11.004

[CIT0096] Zampini, M., & Spence, C. (2010). Assessing the role of sound in the perception of food and drink. *Chemosensory Perception*, 3(1), 57–67. 10.1007/s12078-010-9064-2

